# Wearable Sensors for Ensuring Sports Safety in Children with Autism Spectrum Disorder: A Comprehensive Review

**DOI:** 10.3390/s25051409

**Published:** 2025-02-26

**Authors:** Ofir Arbili, Lior Rokach, Seffi Cohen

**Affiliations:** Software and Information Systems Engineering, Ben-Gurion University, David Ben-Gurion Blvd. 1, Beer Sheva 84105, Israel; arbili@post.bgu.ac.il (O.A.);

**Keywords:** wearable sensors, autism spectrum disorder, IoT, sports safety, physiological monitoring, ASD

## Abstract

Children with Autism Spectrum Disorder (ASD) often face unique risks during sports activities due to challenges such as motor coordination difficulties, sensory sensitivities, and communication impairments. This paper provides a comprehensive review of the use of wearable sensor technologies to enhance the safety and participation of children with ASD in sports. Utilizing a systematic approach, we analyze 144 papers identified through advanced search methodology. Our findings reveal that wearable sensors can monitor physiological signals like heart rate variability and electrodermal activity and biomechanical signals such as movement patterns to detect early signs of distress, anxiety, or potential injury. The integration of these technologies into sports settings for children with ASD presents significant potential for improving safety, reducing participation barriers, and enhancing overall well-being. Key findings indicate that while the application of wearable sensors in this context is still emerging, early results are promising. However, challenges remain regarding device usability, data privacy, and the need for further research to validate the effectiveness of these technologies in real-world sports environments. This review highlights the importance of interdisciplinary collaboration among researchers, technology developers, educators, and caregivers to develop and implement wearable sensor solutions that are tailored to the unique needs of children with ASD, thereby promoting safer and more inclusive sports participation.

## 1. Introduction

Autism Spectrum Disorder is a neurodevelopmental condition characterized by challenges in social interaction, communication, and repetitive behaviors. Children with ASD often experience difficulties in sports participation due to a combination of motor coordination challenges, sensory sensitivities, and difficulties in understanding and responding to social cues [1]. These challenges not only limit their ability to engage in physical activities but also increase the risk of injuries and emotional distress during sports activities.

Wearable sensors offer a novel approach to mitigating these risks by continuously monitoring physiological and biomechanical signals such as heart rate variability—a marker of stress and anxiety—and movement patterns that may indicate an impending injury [2,3]. These real-time data enable timely interventions to prevent harm and promote inclusive sports participation.

The use of wearable sensors to monitor physiological and behavioral parameters in individuals with ASD has been the subject of increasing research interest, as reflected in several review studies [4,5,6,7]. Furthermore, the application of wearable technology in sports and exercise science is also well documented, with numerous reviews focusing on performance enhancement and injury prevention in athletes [8,9,10]. However, to the best of our knowledge, no comprehensive review has specifically addressed the integration of wearable sensor technologies for ensuring sports safety for children with ASD, explicitly considering their unique needs and challenges in sports environments.

Recent years have witnessed significant advancements in wearable sensor technologies, expanding their applications beyond human health and sports. Notably, there is growing interest in the use of wearable sensors for animal monitoring, demonstrating the versatility and potential of these technologies in diverse contexts [11,12].

This paper presents a comprehensive review of the current state of research on wearable sensor technologies for enhancing sports safety among children with ASD. Through a systematic analysis of the literature, facilitated by advanced search methodologies, we aim to synthesize the existing knowledge, identify gaps, and propose future research directions. The rationale for this comprehensive approach is to provide a thorough understanding of the potential and limitations of wearable sensors in this specific application, thereby informing the development of more effective and tailored safety interventions.

## 2. Background and Literature Review

### 2.1. Foundational Concepts

ASD is a complex neurodevelopmental disorder characterized by persistent deficits in social communication and social interaction across multiple contexts, as well as restricted, repetitive patterns of behavior, interests, or activities [4]. The prevalence of ASD has been increasing globally, highlighting the need for innovative approaches to support individuals with ASD in various aspects of life, including sports participation [13].

Sports and physical activity are crucial for the physical and mental health of all children, including those with ASD. Participation in sports can improve motor skills, enhance social interactions, and reduce challenging behaviors [1]. However, children with ASD often face significant barriers to safe and successful sports participation. These barriers include motor coordination difficulties, sensory sensitivities, social communication challenges, and an increased risk of injury due to difficulties in understanding and following safety instructions [14].

Wearable sensor technologies have emerged as a promising tool for addressing some of these challenges. These devices can monitor a wide range of physiological and biomechanical signals, such as heart rate, skin temperature, movement patterns, and impact forces [3]. By providing real-time data on these parameters, wearable sensors can help identify early signs of distress, anxiety, or physical strain, allowing for timely interventions to prevent injuries and improve the overall sports experience for children with ASD [7].

### 2.2. Insights from the Literature

The application of wearable sensors in the context of ASD and sports safety has been explored in several studies. For instance, research has shown that wearable sensors can effectively monitor physiological stress indicators, such as heart rate variability and electrodermal activity, in individuals with ASD [2,5]. This capability can be particularly valuable in sports settings, where children with ASD may experience increased stress and anxiety due to the unpredictable nature of the environment and social interactions [15].

Furthermore, wearable sensors have been used to analyze movement patterns and detect atypical motor behaviors in children with ASD [16]. This information can be used to identify potential safety risks, such as falls or collisions, and to develop targeted interventions to improve motor skills and coordination [3].

### 2.3. Timeline of Research

**Figure 1:**
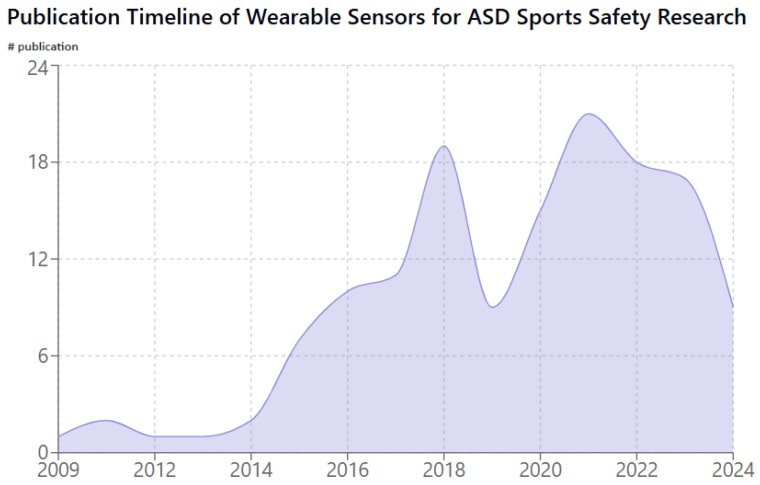
Temporal distribution of research publications on wearable sensors for ASD sports safety from 2009 to 2024. Data were obtained using the Undermind.ai platform with the following base prompt: “The application of wearable sensors to ensure general sports safety for children with Autism Spectrum Disorder”. The number of publications per year represents reports identified by Undermind.ai as relevant to this search query.

A timeline of key developments in this field is presented below. As shown in Figure 1, the temporal distribution of research publications on wearable sensors for ASD sports safety spans from 2009 to 2024. The chart demonstrates a significant increase in research activity starting from 2015, with a peak publication volume of 21 papers in 2021, followed by sustained high activity through 2023. This recent growth trend suggests increasing interest in this field, with 2024 already showing activity in its early months. Notably, there is a dip in publication numbers around 2019 compared to 2018. It is possible that this represents a natural fluctuation in research output within this specific niche area, or it could be due to variations in database coverage for that particular year. However, the overall trend clearly indicates a growing body of literature in this field, especially from 2015 onwards.

The research on wearable sensors for ASD and sports safety has evolved over time, with early studies focusing on basic feasibility and proof of concept [16], and more recent work exploring the integration of multiple sensors and advanced data analytics techniques [4,5]. A timeline of key developments in this field is presented below.

- **2010–2015**: Initial explorations of wearable sensors for monitoring physiological and behavioral parameters in individuals with ASD [17].
- **2016–2018**: Development of sensor-based systems for detecting specific behaviors, such as self-stimulatory movements or critical events like falls, in children with ASD [16,18,19].
- **2019–2021**: Integration of multiple sensors and IoT technologies to create more comprehensive monitoring systems for individuals with ASD [20,21].
- **2022–Present**: Focus on real-world applications and the development of personalized, adaptive interventions based on sensor data analysis [4,5,22].

## 3. Methodology

This comprehensive review is augmented by the Undermind.ai [23] platform, a multiagent system designed for systematic academic literature search and analysis. The platform employs language models as reasoning engines and classifiers within a structured exploration process to identify and evaluate relevant research papers.

### 3.1. Search Platform and Process

The Undermind.ai platform executes searches through four key steps:

1. **Basic search:** The system identifies candidate papers using a custom algorithm that combines semantic vector embeddings, citations, and language model reasoning.
2. **Relevance classification:** Papers are classified into three categories (highly relevant, closely related, or ignorable) using GPT-4 [24] as the language model classifier, evaluating papers based on their full text against the search criteria.
3. **Adaptation and exploration:** The algorithm adapts its search strategy based on discovered content, mimicking human discovery processes to ensure comprehensive coverage.
4. **Comprehensiveness estimation:** The system tracks discovery rates of relevant papers to determine search saturation, following an exponential discovery curve that indicates when nearly all relevant papers have been found.

### 3.2. Search Implementation

We initiated the search using the following base prompt:

“The application of wearable sensors to ensure general sports safety for children with Autism Spectrum Disorder.”

This search query was processed by the platform’s multi-agent system, which examined the full text of open-access papers and those in the ArXiv database. The system’s language model classifier assessed the relevance of each candidate paper to our specific research focus on wearable sensors for ASD sports safety.

### 3.3. Results Processing

The search process identified 144 papers, estimated to represent approximately 84.8% of all relevant papers existing in the database on this topic. This estimation is based on the platform’s exponential discovery curve analysis, which tracks the rate of finding new relevant papers during the search process.

Papers were systematically classified according to their relevance using the following criteria:

- **Highly relevant:** Papers directly addressing wearable sensor technologies for sports safety in children with ASD;
- **Closely related:** Papers discussing either wearable sensors in other contexts for ASD or sports safety technologies without specific focus on ASD;
- **Ignorable:** Papers not substantively contributing to the research focus.

The platform’s classifier demonstrated high accuracy in categorization, with a verified accuracy rate of approximately 98% for highly relevant papers, ensuring reliable content selection for our review.

## 4. Results

The systematic search and selection process, facilitated by the undermine.ai platform, resulted in the inclusion of 144 papers for this comprehensive review. The findings from these studies are summarized below, highlighting key patterns, gaps, and inconsistencies in the literature. In the following subsections, we provide an overview of the extracted data from a subset of the most relevant studies.

### 4.1. Summary of Findings

Recent studies show increasing interest in wearable sensors for improving sports safety and well-being in children with ASD. The literature review highlights six key themes.

**Detection of Challenging Behaviors:** Wearable sensors help identify self-injurious behavior and aggression, enabling timely intervention. For instance, Rad et al. demonstrated that wearable sensors could identify challenging behaviors in children with ASD in real-world settings, which can help in timely intervention to prevent injuries. Rad et al.’s advanced laboratory prototypes, combining physiological and movement sensors, showed promising results in identifying challenging behaviors, though the system’s complexity is considerable [25].

**Prediction of Aggression:** Goodwin et al. showed that wearable biosensors could predict aggression in youth with ASD up to one minute before it occurs, allowing for preemptive measures to be taken to ensure safety during sports activities. Goodwin et al. utilized specialized biosensor systems in clinical settings, achieving AUC values of 0.71–0.84 for aggression prediction, but real-world predictive accuracy needs further investigation [26].

**Physiological Monitoring:** Many studies focused on the use of wearable sensors to monitor physiological signals, such as heart rate, heart rate variability (HRV), and electrodermal activity (EDA), in children with ASD [2,5,27]. These studies generally found that wearable sensors could reliably capture physiological changes associated with stress, anxiety, and emotional arousal in this population. Studies on this topic used both laboratory models and commercial devices, such as heart rate monitors and EDA sensors, with varying performance levels and limitations like motion artifacts.

**Behavioral Monitoring:** Several studies explored the use of accelerometers, gyroscopes, and other motion sensors to detect and classify behaviors relevant to safety, such as falls, self-stimulatory movements, and wandering [3,16,28]. These studies demonstrated the feasibility of using wearable sensors to monitor and analyze movement patterns in children with ASD, although the accuracy of behavior classification varied across studies. Behavioral monitoring studies primarily used laboratory-grade accelerometers and gyroscopes in prototypes, demonstrating feasibility in controlled settings, but real-world accuracy needs more evaluation.

**Environmental Monitoring:** Some studies incorporated environmental sensors, such as GPS, proximity sensors, and temperature sensors, into wearable systems to provide contextual information and enhance safety monitoring [20,22,29]. These studies highlighted the potential of integrating multiple sensor modalities to create a more comprehensive understanding of the child’s environment and potential safety risks. Environmental monitoring studies used commercial GPS modules and integrated systems, showing effective GPS tracking outdoors, but battery life and indoor accuracy remain limitations.

**Real-time Alerts and Interventions:** Several studies developed systems that provided real-time alerts or feedback to caregivers based on sensor data analysis [18,30,31]. These systems aimed to enable timely interventions to prevent injuries, manage challenging behaviors, or provide support during stressful situations. Real-time alert systems were mainly prototype-based, using microcontrollers for alert generation, demonstrating feasibility but highlighting wear tolerance and hardware robustness as challenges.

As summarized in Table 1, several key themes emerged from the literature on wearable sensor technologies for ensuring sports safety in children with ASD. These themes range from conventional physiological and behavioral monitoring to advanced applications such as detecting challenging behaviors and predicting aggression, thereby demonstrating the multifaceted approaches currently explored in the field.

### 4.2. Patterns and Trends

**Figure 2:**
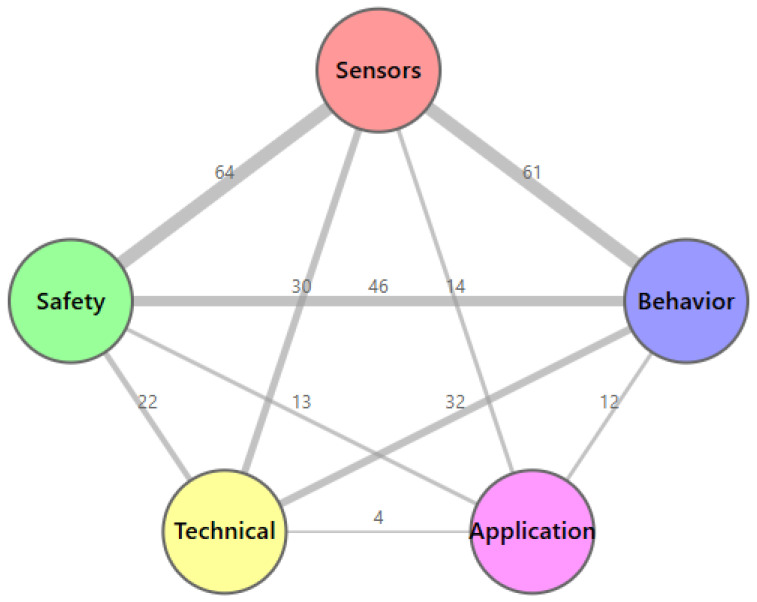
Research focus network showing the interconnections between major themes in the literature. Line thickness represents the strength of connection based on co-occurrence in publications. The strongest relationships exist between sensors and safety (64 co-occurrences) and sensors and behavior monitoring (61 co-occurrences), highlighting the field’s emphasis on using sensor technology for safety monitoring and behavioral assessment.

Several key trends emerged in the use of wearable sensors for ASD sports safety. As illustrated in Figure 2, the strongest connections exist between sensors and safety, as well as sensors and behavior monitoring, underscoring the field’s emphasis on real-time monitoring and intervention.

**Increasing Complexity of Sensor Systems:** Early studies often focused on single-sensor applications, such as using accelerometers to detect falls [16]. More recent studies trend towards integrating multiple sensor modalities, including physiological, behavioral, and environmental sensors, to create more comprehensive monitoring systems [4,5,20].

**Growing Use of Machine Learning:** Many recent studies have employed machine learning algorithms to analyze sensor data and detect patterns indicative of safety risks or emotional distress [31,32,33]. These approaches have shown promise in improving the accuracy and personalization of wearable sensor systems.

**Shift Towards Real-World Applications:** While early studies often focused on feasibility testing and proof of concept in controlled settings [17], more recent work has emphasized the importance of real-world validation and the development of systems that can be practically implemented in everyday environments [4,5,6].

**Focus on User-Centered Design:** Several studies have highlighted the importance of user-centered design principles in the development of wearable sensor technologies for children with ASD [7,28,34]. This includes considerations such as comfort, wearability, sensory sensitivities, and the involvement of caregivers and children in the design process.

### 4.3. Gaps and Inconsistencies

Despite progress, key gaps remain in the literature:

**Limited Focus on Sports Safety:** While many studies addressed general safety concerns for children with ASD, few specifically focused on the application of wearable sensors in sports or physical activity contexts [3]. This represents a significant gap, given the unique safety challenges faced by children with ASD during sports participation.

**Lack of Standardization:** There is a lack of standardization in terms of sensor types, data collection protocols, and outcome measures across studies. This makes it difficult to compare findings and draw definitive conclusions about the effectiveness of different wearable sensor technologies.

**Small Sample Sizes:** Many studies included in this review had small sample sizes [18,27,35], which limits the generalizability of the findings. Larger-scale studies are needed to establish the effectiveness and feasibility of wearable sensor technologies for diverse populations of children with ASD.

**Limited Long-Term Data:** Most studies collected data over relatively short periods, ranging from a few hours to several weeks [36,37,38]. Longitudinal studies are needed to assess the long-term usability, acceptability, and impact of wearable sensor technologies on sports safety and participation in children with ASD.

### 4.4. Open Questions

Several open questions emerge from the literature:

**Device Usability:** How can wearable sensor devices be designed to maximize comfort, minimize sensory sensitivities, and ensure long-term usability for children with ASD in sports settings?

**Data Privacy:** What are the ethical considerations and best practices for collecting, storing, and sharing sensitive physiological and behavioral data from children with ASD, particularly in the context of sports and physical activity?

**Cost Barriers:** How can the cost of wearable sensor technologies be reduced to make them more accessible to families, schools, and sports organizations serving children with ASD?

**Integration with Existing Practices:** How can wearable sensor data be effectively integrated into existing sports safety protocols, coaching practices, and individualized education plans (IEPs) for children with ASD?

**Personalization:** How can machine learning algorithms and data analytics techniques be used to personalize wearable sensor systems and interventions based on the unique needs and characteristics of individual children with ASD?

In the following discussion, we interpret these findings in light of existing theoretical frameworks and practical constraints, highlighting technical, practical, and ethical considerations for the adoption of wearable sensor technologies in sports settings for children with ASD.

## 5. Discussion

The results of this comprehensive review highlight the significant potential of wearable sensor technologies to enhance the safety and well-being of children with ASD, particularly in the context of sports and physical activity. The findings also underscore the need for further research and development to address the identified gaps and open questions.

### 5.1. Interpretation of Results

The studies included in this review demonstrate that wearable sensors can effectively monitor a range of physiological, behavioral, and environmental parameters relevant to the safety of children with ASD. Physiological monitoring, particularly of Heart Rate Variability (HRV) and Electrodermal Activity, has shown promise in detecting early signs of stress, anxiety, and emotional dysregulation [2,5,27]. This capability is particularly important in sports settings, where children with ASD may experience heightened sensory and social challenges [15]. The correlation of HRV and EDA with actual physical or emotional distress in real-world sports scenarios requires further consideration. **Correlation with Emotional Distress:** HRV, particularly the RMSSD (Root Mean Square of Successive Differences between heartbeats) and HF (High Frequency) components, is often inversely related to psychological stress and anxiety. Lower HRV generally indicates higher stress. EDA, reflecting changes in sweat gland activity, increases with emotional arousal and sympathetic nervous system activation, which is associated with stress, anxiety, and excitement. In sports scenarios for children with ASD, heightened sensory input, social demands, and the pressure to perform can trigger emotional distress. Elevated EDA and reduced HRV in these contexts could indeed reflect this distress. For example, a child experiencing sensory overload during a noisy team sport might exhibit increased EDA and decreased HRV concurrently with observable signs of distress like agitation or withdrawal. **Correlation with Physical Distress and Safety Risks:** While HRV and EDA are primarily indicators of emotional and physiological stress, they can also indirectly correlate with physical distress and safety risks in sports. For instance, significant physical exertion or dehydration can also impact HRV and EDA. Moreover, emotional distress can lead to behaviors that increase physical risk, such as impulsive actions or reduced awareness of surroundings. Therefore, while not direct measures of physical injury risk, changes in HRV and EDA can serve as early warning signs that a child might be approaching a state where they are more vulnerable to accidents or physical distress.

Behavioral monitoring using accelerometers, gyroscopes, and other motion sensors has also been shown to be feasible and effective in detecting atypical movement patterns, falls, and other safety-relevant events [3,16,28]. This information can be used to alert caregivers to potential risks and inform interventions to improve motor skills and coordination.

The integration of environmental sensors, such as GPS and proximity sensors, further enhances the capabilities of wearable systems by providing contextual information about the child’s surroundings [20,22,29]. This is particularly relevant in sports settings, where children may be at risk of wandering, collisions, or exposure to unsafe environmental conditions.

The use of machine learning algorithms to analyze sensor data and detect patterns indicative of safety risks or emotional distress is a growing trend in this field [31,32,33]. These techniques have the potential to improve the accuracy, sensitivity, and personalization of wearable sensor systems, enabling more effective and timely interventions.

### 5.2. Technical, Practical, and Ethical Considerations

**Figure 3:**
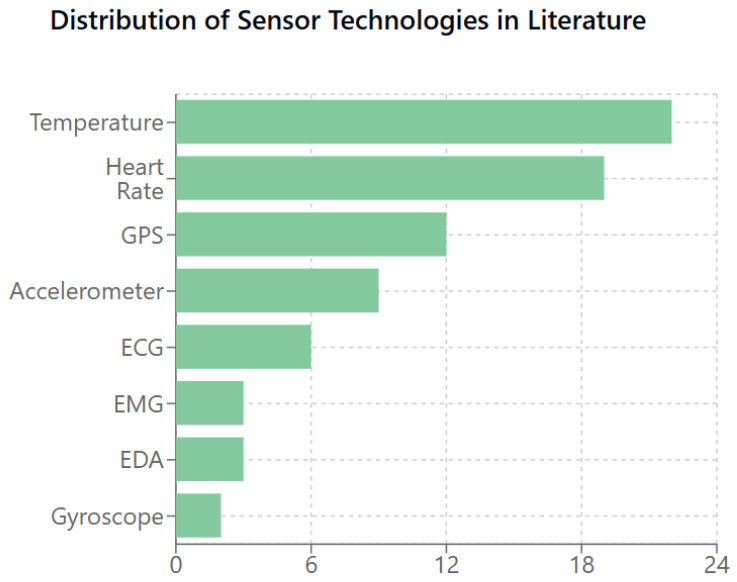
Distribution of sensor technologies discussed in the literature. Temperature sensors (22 mentions) and heart rate monitoring devices (19 mentions) emerge as the most frequently studied technologies, followed by GPS (12 mentions) and accelerometers (9 mentions). This distribution reflects the field’s focus on physiological monitoring and location tracking for safety applications.

As shown in Figure 3, temperature sensors and heart rate monitors are most frequently mentioned in the literature, reflecting an emphasis on physiological and environmental monitoring for safety applications.

The implementation of wearable sensor technologies for sports safety in children with ASD raises several important technical, practical, and ethical considerations.

**Technical:**

- **Sensor Accuracy and Reliability:** Accurate and reliable sensor data are essential for wearable systems, particularly when monitoring the diverse and atypical movement patterns of children with ASD. Variability from motion artifacts, signal noise, and individual differences requires advanced strategies to enhance detection precision. Recent innovations address these challenges through several approaches:
  – **Signal Processing:** Advanced filtering methods (e.g., Kalman filtering, wavelet transforms, adaptive filtering) help mitigate noise and motion artifacts in accelerometer and gyroscope data, isolating subtle movement deviations essential for accurate detection [39].
  – **Sensor Fusion:** Integrating data from multiple sensors—such as accelerometers, gyroscopes, magnetometers, and physiological monitors—provides a robust and comprehensive movement profile. Fusion algorithms (e.g., complementary filters, extended Kalman filters) reduce noise and enhance recognition accuracy.
  – **Machine Learning for Pattern Recognition:** Employing advanced machine learning models, including ensemble methods and test-time augmentation [40,41], improves detection of both typical and atypical movements. Training on extensive datasets that capture the unique movement signatures of children with ASD helps to significantly reduce false positives.
  – **Personalized Calibration and Adaptation:** Systems that calibrate to individual baseline movement patterns enable personalized sensitivity and specificity. Adaptive algorithms continuously update these models, accommodating changes in a child’s movement over time.
  – **Improved Sensor Hardware:** Advances in sensor design—such as higher sampling rates and lower noise components—directly enhance data fidelity. More robust hardware minimizes the effects of motion artifacts, bolstering overall system reliability.
  Collectively, these improvements offer a more precise, real-time understanding of a child’s movements, thereby contributing to safer environments and more effective injury prevention strategies [5].
- **Data Integration and Fusion:** Integrating data from multiple sensors and modalities can provide a more comprehensive and nuanced understanding of the child’s state and environment. However, effective data fusion techniques are needed to combine and interpret these diverse data streams [20].
- **Real-time Processing and Feedback:** For wearable sensors to be effective in preventing injuries and managing challenging behaviors during sports, real-time processing and feedback capabilities are essential. This requires efficient algorithms and low-latency communication between sensors, processing units, and caregiver devices [18,30,31].

**Practical:**

- **Usability and Acceptability:** Wearable devices for children with ASD must be comfortable, discreet, and simple to use. Given these children’s heightened sensory sensitivities, devices should employ hypoallergenic, soft, breathable materials, be lightweight and adjustable, and offer customizable sensory feedback (e.g., subtle vibration or low-stimulation visual cues). Incorporating user-centered design-engaging models for children, caregivers, and therapists throughout development is essential to minimize sensory overload and maximize overall usability [7,28,34].
- **Cost and Accessibility:** The cost of wearable sensor technologies can impede their adoption, especially among families with limited resources and schools serving diverse populations. To enhance accessibility, several cost reduction strategies should be considered: **Open-Source Hardware and Software Solutions**, **Using Affordable and Versatile Sensors** such as smartphones [18] and **Multi-Tiered Designs**. Recognizing that high-quality, sensory-sensitive materials may be costly, a multi-tier product strategy (offering both basic and premium options) can accommodate varying budgets and user needs. Improving accessibility also means ensuring that devices are user-friendly for diverse populations, offering multilingual training and support as well as implementing culturally sensitive strategies [6].
- **Training and Support:** Caregivers, parents, and educators need training and support to effectively use wearable sensor technologies and interpret the data they provide. This includes understanding how to respond to alerts, manage challenging behaviors, and integrate sensor data into individualized education and safety plans [42].

**Ethical:**

- **Privacy and Data Security:** The collection and storage of sensitive physiological and behavioral data from children with ASD raises significant privacy and data security concerns. Privacy protocols must be in place to ensure that data are collected, stored, and shared ethically and in compliance with relevant regulations [7].
- **Informed Consent:** Obtaining informed consent from parents is essential before implementing wearable sensor technologies. This includes clearly explaining the purpose of data collection, the potential benefits and risks, and the procedures for data management and sharing [43].
- **Equity and Access:** Efforts should be made to ensure that wearable sensor technologies are accessible to all children with ASD who could benefit from them, regardless of their socioeconomic status, geographic location, or the severity of their condition. This may require addressing disparities in access to technology, internet connectivity, and specialized support services [6].

### 5.3. Integration with Sports Safety Protocols and IEPs

Integrating wearable sensor data into existing sports safety protocols and Individualized Education Plans (IEPs) offers a significant opportunity to enhance the safety and inclusion of children with ASD in sports.

**Integration with Sports Safety Protocols:** Wearable sensor data can provide real-time insights that can be directly incorporated into sports safety protocols. For example:

- **Real-time Monitoring and Alert Systems:** Data from physiological sensors can be used to monitor a child’s stress or anxiety levels during sports activities. If these levels exceed predefined thresholds, automated alerts can be sent to coaches or supervisors, prompting them to check on the child, adjust the activity, or provide a break. This proactive approach can help prevent meltdowns or distress situations before they escalate into safety risks;
- **Biomechanical Data for Injury Prevention:** Wearable motion sensors can track movement patterns and detect potentially risky biomechanics that could lead to injuries. These data can inform coaches about a child’s movement style, highlighting areas where they might be vulnerable. Coaches can then use this information to tailor training, provide specific feedback on technique, and modify activities to reduce the risk of injury. For instance, if a sensor detects an unusual gait pattern during running, it could indicate fatigue or improper form, prompting a coach to intervene.
- **Environmental Context for Safety Adjustments:** Integrating environmental sensor data, such as GPS location, with physiological and biomechanical data can provide a comprehensive view of safety. For example, if a child with ASD is playing in a large field, GPS data combined with physiological data indicating rising anxiety could suggest they are becoming overwhelmed by the open space or are wandering too far from supervision. Protocols can then be in place to ensure closer supervision or a change of environment.

To effectively integrate wearable sensor data into sports safety protocols, it is crucial to consider the following aspects.

- **Establish Clear Protocols and Response Plans:** Develop specific, actionable protocols based on different sensor data patterns. This includes defining thresholds for alerts, outlining steps for intervention, and ensuring that coaches and staff are trained to understand and respond appropriately to sensor-generated alerts.
- **Ensure Data Privacy and Security:** Implement robust systems to protect the privacy of sensor data and ensure secure data handling, storage, and transmission, adhering to ethical guidelines and regulations.
- **Maximize Comfort:** Wearable devices must be comfortable, non-stigmatizing, and easy to use for both children with ASD and their caregivers. This is particularly critical due to the sensory sensitivities often experienced by children with ASD. Design considerations must prioritize minimizing sensory overload and maximizing comfort.

**Integration with IEPs:** Wearable sensor data can also be a valuable tool for personalizing IEPs for children with ASD, particularly in the domain of physical education and sports participation.

- **Data-Driven Goal Setting:** Sensor data can provide objective measures of a child’s responses to different sports activities. These data can be used to set realistic and measurable IEP goals related to physical activity, emotional regulation during exercise, and skill development. For example, an IEP goal might be to increase participation in team sports while maintaining heart rate variability within a healthy range, as monitored by a wearable sensor.
- **Monitoring Progress and Adapting Interventions:** Wearable sensors allow for continuous monitoring of a child’s progress towards their IEP goals. Data collected over time can show improvements in physiological responses to exercise, changes in movement patterns, or reductions in stress indicators during sports. This longitudinal data can inform IEP reviews and adjustments, ensuring that interventions remain effective and tailored to the child’s evolving needs.
- **Personalized Activity Recommendations:** By analyzing sensor data in conjunction with a child’s IEP goals and sensory profile, personalized recommendations for sports and physical activities can be generated. For example, if a child’s IEP emphasizes social interaction and sensor data indicate they remain calm and engaged during structured team activities, the IEP team might recommend increasing participation in such activities. Conversely, if data show high stress during noisy, fast-paced games, the IEP might suggest more individual or low-sensory sports options.

Successful integration of wearable sensor data into IEPs requires the following:

- **Collaboration Between IEP Teams and Technology Experts:** It is necessary to ensure that IEP teams include members who understand wearable sensor technology and data interpretation, or to provide training to existing team members.

By thoughtfully integrating wearable sensor data into sports safety protocols and IEPs, we can create more supportive, safe, and inclusive sports environments that enable children with ASD to fully participate and benefit from physical activity.

### 5.4. Limitations and Future Work

This comprehensive review has several limitations that should be considered when interpreting the findings. First, the review focuses primarily on studies identified through the undermine.ai platform, which may have resulted in the exclusion of some relevant studies indexed in other databases.

Second, the quality and rigor of the included studies vary considerably. Many studies have small sample sizes, lack control groups, or rely on short-term data collection periods [18,27,35]. This limits the generalizability of the findings and the strength of the conclusions that can be drawn.

Third, the rapid pace of technological development in the field of wearable sensors means that some of the studies included in this review may be outdated. Newer sensor technologies and data analysis techniques may offer improved performance and capabilities compared to those described in the older studies.

Despite these limitations, this review provides a comprehensive overview of the current state of research on wearable sensors for sports safety in children with ASD and identifies several important directions for future work:

1. **Conducting larger-scale, longitudinal studies** to assess the long-term effectiveness, usability, and acceptability of wearable sensor technologies in real-world sports settings. These studies should involve diverse populations of children with ASD and collect data on a range of outcome measures, including injury rates, physical activity levels, emotional regulation, and social participation;
2. **Developing and validating sport-specific algorithms** for detecting safety risks and predicting challenging behaviors based on sensor data collected during various physical activities. These algorithms should be tailored to the unique characteristics of different sports and the specific needs of children with ASD;
3. **Exploring the integration of wearable sensors with other technologies**, such as virtual reality, augmented reality, and LLMs, to create more immersive, interactive, and personalized sports training and safety interventions for children with ASD;
4. **Integrating wearable sensors for other activities** beyond traditional sports to further enhance the safety and engagement of children with ASD. Expanding the application of these technologies to recreational, educational, and therapeutic activities could provide a more holistic approach to promoting their physical and emotional well-being. Furthermore, integrating these sensors into comprehensive sports safety systems can ensure a tailored approach to the diverse needs of ASD children across different activities;
5. **Investigating the ethical, legal, and social implications** of using wearable sensors with children with ASD, particularly in the context of sports. This includes addressing issues such as data privacy, informed consent, equity of access, and the potential for stigmatization or over-reliance on technology;
6. **Establishing best practices and guidelines** for the development, implementation, and evaluation of wearable sensor technologies for sports safety in children with ASD. This may involve creating standardized protocols for data collection, analysis, and reporting, as well as developing training programs for caregivers, parents, and educators.

## 6. Conclusions

This comprehensive review examines the application of wearable sensor technologies to enhance the general sports safety of children with ASD. The analysis reveals a growing body of research on the use of wearable sensors to monitor physiological, behavioral, and environmental parameters in this population.

The major insights from this review suggest that wearable sensors hold significant potential to improve sports safety for children with ASD by enabling real-time monitoring of stress indicators, detecting atypical movement patterns, and providing contextual information about the environment. The integration of multiple sensor modalities and the use of machine learning algorithms have shown promise in enhancing the accuracy and personalization of these systems.

However, the review also highlights several limitations and gaps in the current literature, including the limited focus on sports-specific applications, the lack of standardization in sensor technologies and data analysis methods, and the need for larger-scale, longitudinal studies to establish the long-term effectiveness and feasibility of these approaches.

Future research should focus on developing and validating sport-specific algorithms, conducting larger-scale studies in real-world environments, exploring the integration of wearable sensors with other emerging technologies, integrating wearable sensors for other activities, and addressing the ethical and practical considerations of using these technologies with vulnerable populations.

## Figures and Tables

**Table 1:** Overview of Key Themes Emerging from the Literature on Wearable Sensor Technologies for Ensuring Sports Safety in Children with ASD, with Representative Studies.

Key Theme	Description	Sensor Implementation Status	Representative Study
Physiological Monitoring	Monitors signals such as heart rate, HRV, and EDA to detect stress or anxiety	Laboratory Models and Commercial Devices Studies utilize both custom-built laboratory setups and commercially available devices	Fioriello et al. [2]
Behavioral Monitoring	Detects movement patterns, falls, and atypical behaviors for safety	Laboratory Models and Prototypes	Bashkaran et al. [3]
Environmental Monitoring	Gathers contextual data (e.g., location, ambient conditions) to enhance safety insights	Commercial and Integrated Systems Employs commercial GPS modules and environmental sensors integrated into custom IoT systems	Wu et al. [22]
Real-time Alerts	Provides immediate feedback and alerts based on sensor data	Commercial and Integrated Systems	Northrup et al. [18]
Detection of Challenging Behaviors	Detects self-injurious behavior and aggression in real-world settings to enable timely intervention	Advanced Laboratory Prototypes Combines physiological (EDA, ECG) and movement sensors in sophisticated lab prototypes	Rad et al. [25]
Prediction of Aggression	Predicts aggression up to one minute in advance for preemptive measures during sports activities	Specialized Biosensor Systems Utilizes wrist-worn biosensors (e.g., Empatica E4) in controlled clinical settings	Goodwin et al. [26]

## Abbreviations

The following abbreviations are used in this manuscript:

LD	Linear Dichroism
ASD	Autism Spectrum Disorder
HRV	Heart Rate Variability
EDA	Electrodermal Activity
IoT	Internet of Things
GPS	Global Positioning System
IEP	Individualized Education Plan
LLM	Large Language Model

## References

[1] Sorgente V., Cohen E.J., Bravi R., Minciacchi D. (2021). Accept. Understand. Then play! The impact of sport and physical activity in autism spectrum disorder. Authorea.

[2] Fioriello F., Maugeri A., D’Alvia L., Pittella E., Piuzzi E., Rizzuto E., Prete Z.D., Manti F., Sogos C. (2020). A wearable heart rate measurement device for children with autism spectrum disorder. Sci. Rep..

[3] Bashkaran K., Radhika K., Selvarasu S., Muthulekshmi M., Valarmathy S. Wireless sensor networks for sports injury management and player safety enhancement. Proceedings of the 2023 International Conference on Self Sustainable Artificial Intelligence Systems (ICSSAS).

[4] Gao X., Yin L., Tian S., Huang Y., Ji Q. (2024). Wearable technology for signal acquisition and interactive feedback in autism spectrum disorder intervention: A review. IEEE Sens. J..

[5] Cano S., Cubillos C., Alfaro R., Romo A., García M., Moreira F. (2024). Wearable solutions using physiological signals for stress monitoring on individuals with autism spectrum disorder (ASD): A systematic literature review. Sensors.

[6] Hernández-Capistrán J., Alor-Hernández G., Marín-Vega H., Bustos-López M., Sanchez-Morales L.N., Sanchez-Cervantes J.L. (2024). Commercial wearables for the management of people with autism spectrum disorder: A review. Biosensors.

[7] Taj-Eldin M., Ryan C., O’Flynn B., Galvin P. (2018). A review of wearable solutions for physiological and emotional monitoring for use by people with autism spectrum disorder and their caregivers. Sensors.

[8] Olsen R.J., Hasan S.S., Woo J.J., Nawabi D.H., Ramkumar P.N. (2025). The fundamentals and applications of wearable sensor devices in sports medicine: A scoping review. Arthroscopy.

[9] Seçkin A.C., Ateş B., Seçkin M. (2023). Review on wearable technology in sports: Concepts, challenges and opportunities. Appl. Sci..

[10] Sun W., Guo Z., Yang Z., Wu Y., Lan W., Liao Y., Wu X., Liu Y. (2022). A review of recent advances in vital signals monitoring of sports and health via flexible wearable sensors. Sensors.

[11] Pan Y., Su X., Liu Y., Fan P., Li X., Ying Y., Ping J. (2024). A laser-engraved wearable electrochemical sensing patch for heat stress precise individual management of horse. Adv. Sci..

[12] Zhou J., Zhou S., Fan P., Li X., Ying Y., Ping J., Pan Y. (2023). Implantable electrochemical microsensors for in vivo monitoring of animal physiological information. Nano-Micro Lett..

[13] Fazana F., Alsadoon A., Prasad P., Costadopoulos N., Elchouemi A., Sreedharan S. Integration of assistive and wearable technology to improve communication, social interaction and health monitoring for children with autism spectrum disorder (ASD). Proceedings of the 2017 IEEE Region 10 Symposium (TENSYMP).

[14] Shevchuk K., Porodko M. (2022). Involvement of children with communication disabilities in sports clubs. Visnyk of the Lviv University. Series Pedagogics.

[15] Pittella E., Piuzzi E., Rizzuto E., Prete Z., Fioriello F., Maugeri A., Sogos C. Wearable heart rate monitoring as stress response indicator in children with neurodevelopmental disorder. Proceedings of the 2018 IEEE International Symposium on Medical Measurements and Applications (MeMeA).

[16] Ann O.C., Theng L.B., Seldon H., Putra F.A. (2015). Critical behavior monitoring for children with special needs in preventing physical injury using kinect. Assistive Technologies for Physical and Cognitive Disabilities.

[17] Min C.-H., Tewfik A. Automatic characterization and detection of behavioral patterns using linear predictive coding of accelerometer sensor data. Proceedings of the 2010 Annual International Conference of the IEEE Engineering in Medicine and Biology.

[18] Northrup C. (2016). Wearable stress sensors for children with autism spectrum disorder with in situ alerts to caregivers via a mobile phone. Iproceedings.

[19] Shi Y., Das S., Douglas S.N., Biswas S. An experimental wearable iot for data-driven management of autism. Proceedings of the 2017 9th International Conference on Communication Systems and Networks (COMSNETS).

[20] Chowdhury U., Chowdhury P., Paul S., Sen A., Sarkar P.P., Basak S., Bhattacharya A. Multi-sensor wearable for child safety. Proceedings of the 2019 IEEE 10th Annual Ubiquitous Computing, Electronics Mobile Communication Conference (UEMCON).

[21] Tomczak M., Wójcikowski M., Pankiewicz B., Łubiński J., Majchrowicz J., Majchrowicz D., Walasiewicz A., Kiliński T., Szczerska M. (2020). Stress monitoring system for individuals with autism spectrum disorders. IEEE Access.

[22] Wu X., Chen J., Cheng Z., Liu X., Wang Y., Liu W., Jiang X. (2024). Physical and mental safety monitoring and protection of children with autism spectrum disorder: Intelligent clothing integrating early warning and rescue. J. Eng. Fibers Fabr..

[23] Undermind.ai (2024). Undermind: AI-Powered Scientific Research Assistant. https://www.undermind.ai/.

[24] Achiam J., Adler S., Agarwal S., Ahmad L., Akkaya I., Aleman F.L., Almeida D., Altenschmidt J., Altman S., Anadkat S. (2023). GPT-4 Technical Report. arXiv.

[25] Rad A.B., Villavicencio T., Kiarashi Y., Anderson C., Foster J., Kwon H., Hamlin T., Lantz J., Clifford G.D. (2025). From Motion to Emotion: Exploring Challenging Behaviors in Autism Spectrum Disorder Through Analysis of Wearable Physiology and Movement. Physiol. Meas..

[26] Goodwin M.S., Mazefsky C.A., Ioannidis S., Erdogmus D., Siegel M. (2019). Predicting Aggression to Others in Youth With Autism Using a Wearable Biosensor. Autism Res..

[27] Nuske H.J., Goodwin M.S., Kushleyeva Y., Forsyth D., Pennington J.W., Masino A.J., Finkel E., Bhattacharya A., Tan J., Tai H. (2021). Evaluating commercially available wireless cardiovascular monitors for measuring and transmitting real-time physiological responses in children with autism. Autism Res..

[28] Amiri A., Peltier N., Goldberg C., Sun Y., Nathan A., Hiremath S.V., Mankodiya K. (2017). Wearsense: Detecting autism stereotypic behaviors through smartwatches. Healthcare.

[29] Feeham S.Y., Akter T., Debnath S., Mia M.S. Risk analysis and support system for autistic children using iot. Proceedings of the 2022 4th International Conference on Sustainable Technologies for Industry 4.0 (STI).

[30] Torrado J.C., Gómez J., Montoro G. (2017). Emotional self-regulation of individuals with autism spectrum disorders: Smartwatches for monitoring and interaction. Sensors.

[31] Deng L., Rattadilok P., Xiong R. A machine learning-based monitoring system for attention and stress detection for children with autism spectrum disorders. Proceedings of the 2021 International Conference on Intelligent Medicine and Health.

[32] Al-Wakeel S., Alhalabi B., Aggoune H.M., Alwakeel M.M. A machine learning based wsn system for autism activity recognition. Proceedings of the 2015 IEEE 14th International Conference on Machine Learning and Applications (ICMLA).

[33] Bringye Z., Kozlovszky M., Farkas K. Are wearable physiological sensors and associated algorithms suitable for detecting or predicting challenging behavior events in patients with autism spectrum disorder? A protocol for a scoping review. Proceedings of the 2022 IEEE 20th Jubilee International Symposium on Intelligent Systems and Informatics (SISY).

[34] Koo S., Gaul K., Rivera S., Pan T., Fong D. (2018). Wearable technology design for autism spectrum disorders. Arch. Des. Res..

[35] D’Alvia L., Pittella E., Fioriello F., Maugeri A., Rizzuto E., Piuzzi E., Sogos C., Prete Z. Heart rate monitoring under stress condition during behavioral analysis in children with neurodevelopmental disorders. Proceedings of the 2020 IEEE International Symposium on Medical Measurements and Applications (MeMeA).

[36] Bell M., Robinson E.B., Day S., Gilbert T.J., Hamilton A., Ward J.A. Lessons on collecting data from autistic children using wrist-worn sensors. Proceedings of the 2022 ACM International Symposium on Wearable Computers.

[37] Nguyen J., Cardy R., Anagnostou E., Brian J., Kushki A. (2021). Examining the effect of a wearable, anxiety detection technology on improving the awareness of anxiety signs in autism spectrum disorder: A pilot randomized controlled trial. Mol. Autism.

[38] Douglas S.N., Shi Y., Das S., Biswas S. (2021). Validation of wearable sensor technology to measure social proximity of young children with autism spectrum disorders. Focus Autism Other Dev. Disabil..

[39] Cohen S., Katz O., Presil D., Arbili O., Rokach L. (2023). Ensemble learning for alcoholism classification using EEG signals. IEEE Sens. J..

[40] Cohen S., Lior E., Bocher M., Rokach L. (2024). Improving severity classification of Hebrew PET-CT pathology reports using test-time augmentation. J. Biomed. Inform..

[41] Cohen S., Cohen-Inger N., Rokach L. (2024). BagStacking: An integrated ensemble learning approach for freezing of gait detection in Parkinson’s disease. Information.

[42] Lee J., Lee T.S., Lee S., Jang J., Yoo S., Choi Y., Park Y. (2021). Development and application of a metaverse-based social skills training program for children with autism spectrum disorder to improve social interaction: Protocol for a randomized controlled trial. JMIR Res. Protoc..

[43] Deng L., Rattadilok P. (2022). A sensor and machine learning-based sensory management recommendation system for children with autism spectrum disorders. Sensors.

